# Opioid and Sucrose Craving Are Accompanied by Unique Behavioral and Affective Profiles after Extended Abstinence in Male and Female Rats

**DOI:** 10.1523/ENEURO.0515-21.2022

**Published:** 2022-04-08

**Authors:** Hannah L. Mayberry, Heather A. DeSalvo, Charlotte C. Bavley, Sara H. Downey, Cindy Lam, Charita Kunta, Ricardo P. Fortuna, Priya H. Doshi, Elizabeth B. Smedley, Mathieu E. Wimmer

**Affiliations:** Department of Psychology and Neuroscience, Temple University, Philadelphia, PA 19122

**Keywords:** addiction, gonadal hormones, heroin, locomotion, self-administration, ultrasonic vocalizations

## Abstract

Incubation of craving refers to the intensification of drug-seeking behavior in response to reward-paired cues over the course of abstinence. In rodents, craving and drug-seeking behaviors have been measured by an increase in lever pressing in the absence of reinforcer availability in response to cue presentations. However, craving in rodents is difficult to define and little is known about the behavioral signatures that accompany increased drug-seeking behavior measured by lever pressing. The affective components of relapse are also important, but understudied in rodents. Hormonal fluctuations influence craving for psychostimulants, but little is known about the impact of the estrous cycle on opioid-seeking behavior. This study sought to delineate the behavioral and affective signatures associated with craving, and to examine the influence of the female estrous cycle on craving. Male and female rats underwent 10 d of intravenous opioid self-administration. Separate cohorts of control rats self-administered oral sucrose, a natural nondrug reward. Cue-induced seeking tests were conducted after 1 or 30d of forced abstinence. These sessions were recorded and scored for overall locomotion, instances of sniffing, grooming, or hyperactivity. Ultrasonic vocalizations (USVs) were also recorded to determine affective profiles that accompany opioid seeking. Although active lever presses and overall locomotion increased unanimously over extended abstinence from heroin and sucrose, a sex- and reinforcer-specific behavioral and affective signature of craving emerged. Furthermore, although the female estrous cycle did not affect taking or seeking, it appears to influence more granular behaviors.

## Significance Statement

Craving was recently added as a diagnostic criterion for substance use disorders. Positive and negative affect may contribute to relapse, although this domain is not fully understood in the context of cue-induced drug seeking in rodents. To enhance the definition of craving and to address potential sex differences, we recorded behaviors and ultrasonic vocalizations (USVs) during early and late abstinence cue-induced relapse tests in male and female rats. The behavioral signatures associated with craving were sex- and reinforcer-specific. In females, the estrous cycle selectively influenced locomotion and hyperactivity during reinforcer seeking. These findings contribute to a better behavioral definition of craving, which lays the foundation to better understand the neural mechanisms underlying craving and drug-seeking behaviors.

## Introduction

Craving is an important hallmark of substance use disorders that was recently added as a diagnostic criterion in the DSM. Re-exposure to drug-paired cues often triggers craving, defined as a compulsion to use the drug. Craving intensifies for several weeks after drug use cessation in humans (Gawin and Kleber, 1986; O’Brien et al., 1992) and rodent models of addiction, referred to as “incubation of craving” (Grimm et al., 2001; Pickens et al., 2011). Incubation of craving is a beneficial and translationally relevant model for studying relapse because (1) it occurs in both humans and rodents; and (2) in rodents, it mirrors the condition in which drug use is discontinued and then individuals reintegrate into a familiar environment after some time away (hospitalization, incarceration, inpatient rehabilitation, etc.). Drug-related stimuli can gain salience over time and induce sensitization, known as incentive sensitization (Berridge and Robinson, 2016). One prominent construct in the addiction field is that incentive sensitization contributes to chronic, compulsive drug seeking, as well as other documented stereotyped behaviors during cue re-exposure (Tindell et al., 2005). This form of reward-related plasticity can last years after cessation of drug use (Paulson et al., 1991; Carr et al., 2020). Thus, incentive sensitization, which posits that drug-paired cues gain salience, likely contributes to incubation of craving where cue re-exposure elicits drug seeking even after prolonged abstinence. Importantly, these phenomena are thought to lead to high relapse rates.

Drug craving is subjective in humans and difficult to model in rodents. In preclinical self-administration paradigms, craving is often measured using the number of responses on a previously reward-associated lever when the reinforcer is not available. However, lever pressing does not capture other potentially important and more nuanced behaviors associated with drug seeking. This study built on a wealth of previous behavioral sensitization studies and incorporated some of the drug-induced stereotypical behaviors in establishing a behavioral signature of opioid craving. Behavioral sensitization refers to documented, drug-induced, predictable (stereotyped) responses that emerge in both humans (Sax and Strakowski, 2001) and rodents (Steketee and Kalivas, 2011), and is not to be confused with incentive sensitization, a theoretical concept that stimuli can induce drug-seeking behavior. These specific stereotyped movements are predictive of drug-induced behavioral and neural plasticity and constitute the foundation of the behavioral signature defined in this study.

Craving for the natural reward sucrose also increases over extended abstinence and was used as a comparison to tease out opioid-specific profiles that accompany cue-induced reinforcer seeking. Sucrose is an important control group because it acts on many of the same neural pathways as drugs of abuse, and yet it is not the intended target of treatments aimed to reduce craving. Given that incubation of craving happens for many drugs of abuse as well as nondrug reinforcers like sucrose, this indicates that, in general, the brain is intensifying the cue-reinforcer association over extended abstinence, regardless of reinforcer. Changes that are consistent across reinforcers suggest overlap in the pathways that drive this process, some of which can be adaptive (as is the case for remembering cues that are predictive of positive natural rewards). However, by isolating drug-specific changes, it is possible to disentangle the pathways that specifically drive maladaptive drug-cue association intensification throughout abstinence. These are promising targets for treating opioid craving without affecting the natural reward system.

Positive and negative affective states are thought to contribute to relapse potential (McKay et al., 1996; Rubin et al., 1996; Witkiewitz and Bowen, 2010; Becker and Koob, 2016) and clinical models of relapse include affective state (Witkiewitz and Marlatt, 2004). However, this domain is not often considered in preclinical models of reinstatement or cue-induced incubation of craving, particularly for opioids. Rodent affective states can be measured using ultrasonic vocalizations (USVs). Low-frequency 22-kHz USVs (18–33 kHz) correspond to negative-valence affective states and are emitted in situations of fear or anxiety. High-frequency 50-kHz USVs (38–90 kHz) correspond to positive-valence affective states and are emitted in response to rewarding situations such as social play and drug or food self-administration (Barker et al., 2015). Ultimately, USVs can elucidate affective components of opioid craving in rats.

There are no sex differences in incubation of heroin craving, as measured by active lever presses (Venniro et al., 2019); however, it is possible that males and females differ in terms of more granular addiction-related behaviors. For example, females show enhanced locomotor sensitization in response to experimenter-delivered methamphetamine (Robinson, 1984) and cocaine (Carr et al., 2020). Females also show greater incentive sensitization following intermittent access cocaine self-administration (Kawa and Robinson, 2019). Limited studies have examined the role of biological sex in USV production. Female High Alcohol Drinking (HAD-1) rats emit more 50-kHz USVs during ethanol access compared with males (Mittal et al., 2019). It is unknown whether there is a sex-specific behavioral or affective signature associated with sucrose or opioid craving that persists into protracted abstinence.

Another important consideration that may influence some of these behaviors is the female estrous cycle. The estrous cycle affects addiction-like behaviors including cocaine conditioned place preference, and incubation of cocaine craving (Paris et al., 2014; Calipari et al., 2017; Nicolas et al., 2019). However, results related to whether the estrous cycle impacts opioid-related behaviors are conflicting. Furthermore, few studies have examined the potential role of the estrous cycle on sucrose self-administration or stereotyped behaviors. We address these gaps in knowledge by including males and females and by tracking the estrous cycle throughout in our assessments of intake, craving, and other addiction-related behaviors.

The overarching goal of this work was to delineate and fine-tune the more subtle changes in behavior and affective state that accompany increased drug seeking in response to cue re-exposure. We aimed to identify behaviors that accompany craving generally. Another objective of this study was to identify drug-specific behavioral correlates of craving that do not generalize to natural rewards. This method provides important insight into characteristics of “normal” (nonpathological) craving behaviors versus those that specifically accompany heroin craving. Furthermore, sucrose comparisons can aid in differentiating the mechanisms of cue-reward associations and memory consolidation broadly from those that govern opioid-paired cue-induced craving. These findings can (1) improve the preclinical model of opioid craving/relapse-like like behavior and (2) lay the groundwork for identification of circuits governing opioid-cue reactivity. Males and females were included to address potential sex differences. All measurements were tracked across the estrous cycle with the goal of identifying whether fluctuations in gonadal hormones affect self-administration, craving, and/or any of the more nuanced behaviors. These findings contribute to a better behavioral definition of craving, and aid in identifying opioid-specific neural circuits involved in craving.

## Materials and Methods

### Animals

For all experiments, male (*n* = 51) and female (*n* = 66) Long–Evans rats were bred in house. All animals used in the studies were 60–180 d old and were pair-housed. Rats were housed in a reverse light-dark cycle colony room with lights off at 9 A.M. and were handled daily for 2–5 min each for at least 5d before the start of any behavioral procedure or test. Experiments were conducted in the dark phase. Animals had *ad libitum* access to water and standard laboratory chow throughout the experiment. All animal procedures were performed in accordance with the Temple University animal care committee’s regulations.

### Heroin self-administration, forced abstinence, and relapse tests

Rats used for intravenous heroin self-administration were anesthetized using an intraperitoneal injection of 80 mg/kg ketamine and 12 mg/kg xylazine before surgery. An indwelling SILASTIC catheter was threaded subcutaneously over the shoulder blade, inserted in the jugular vein, and sutured in place. The catheter routed to a mesh back mount platform (Strategic Applications Inc.), which was sutured below the skin between the shoulder blades. Catheters were flushed daily with 0.2 ml of timentin (0.93 mg/ml) dissolved in heparinized saline to prevent clogging. Following the 7-d recovery period, rats were placed in operant chambers and allowed to lever press on a fixed ratio 1 (FR1) schedule for heroin infusions (0.10 mg/kg/infusion over 5 s). Responses on the “active lever” resulted in one infusion, accompanied by a 5-s light cue and a subsequent 20-s timeout period during which the house light was off and lever presses were recorded but had no contingent drug infusion. Sessions began at the start of the animals’ dark cycle (9 A.M.). Heroin self-administration sessions followed an intermittent access paradigm, which included 3h of “drug-available” time, followed by a 15-min window of “no-drug available.” This procedure repeated three times per session, resulting in a total of 9h of drug availability, and was performed daily on 10 consecutive days. Heroin self-administering rats were tested behaviorally for signs of craving during early (day 1) and late (day 30) abstinence using a within-subjects design. Within-subjects relapse tests (“cue tests”) began at the start of the animals’ dark cycle and were 30 min long under extinction conditions, meaning that active lever presses resulted in cue light presentation, but no contingent reward. Throughout abstinence (one or 30 d), rats were housed in their home cage, were not re-exposed to the self-administration chambers, and were handled regularly.

### Sucrose self-administration, forced abstinence, and relapse tests

Separate cohorts of male and female rats self-administered sucrose. This natural, nondrug reward acts as an important control group in establishing baseline behaviors that change over 30 d of forced abstinence. Unsurgerized drug-naive rats responded on the “active lever” for one sucrose pellet (FR1 schedule) that was accompanied by a 5-s light cue, and a subsequent 20-s timeout period as previously described. Sucrose self-administration began at the start of the animals’ dark cycle and occurred for 2 h daily on 10 consecutive days. At the end of one or 30 d of forced abstinence (as described above), male and female rats were tested for sucrose seeking for 1 h.

### Recording relapse tests and scoring for stereotyped behaviors

Each cue test was video recorded for off-line scoring by investigators blind to condition. Videos were recorded in 5 min increments via SD card dashcams (Pruveeo) attached to the top of each sound-attenuating chamber to provide an aerial view of the operant box floor. Raw footage was then edited together using Adobe Premiere Pro (Adobe Inc.) to create one continuous video.

Scored behaviors included horizontal locomotion and stereotypies. These were tracked using an online counter and timer. To measure horizontal locomotion, a two by two grid was superimposed over the floor of the operant chamber displayed on the computer screen in the recording. The grid was measured to ensure that it was ∼15 × 15 cm and that each quadrant was of equal size. Scorers then counted each “beam break,” or instance in which the rat moved from one quadrant of the chamber to another by crossing one of the grid lines with its head and shoulders. Time spent grooming was quantified by the amount of time an animal spent licking, pawing, or scratching itself. Time spent sniffing was quantified by the amount of time that an animal’s nose was in contact with the floor or walls of the operant box, either while stationary, or while mobile. The number of hops or darts across the operant chamber quantified instances of hyperactivity.

### Audio recording and scoring USVs

USVs were collected from a subset of rats during cue tests. Ultrasonic microphones from Dodotronic (Ultramic 200K) were placed atop the self-administration chamber and positioned inside a foam noodle to secure them in an upright position. Microphones were plugged into HP computers and USV recordings were obtained using Raven Pro 1.5 β at a 192,000-Hz sampling rate in 10-min segments. WAVE files were run through DeepSqueak, a MATLAB-based USV detection program.

The Short Rat Call neural network was used to detect 50-kHz USVs. Calls were accepted or rejected based on whether they met frequency criteria (38–90 kHz) and duration criteria (5 ms or longer). Calls were differentiated from background noise by visual confirmation of a clear shape on the spectrogram. The Long Rat Call network was used to detect 22-kHz USVs within the range of 18–33 kHz. Trained scorers counted the number of USVs during the duration of the cue test. If needed, calls were verified via playback in DeepSqueak. Calls in the 18- to 33-kHz range were termed “22-kHz USVs” and were interpreted as a negative-valence affective state. Calls in the 38- to 90-kHz range were termed “50-kHz USVs” and were interpreted as a positive-valence affective state (Barker et al., 2015).

### Gonadal hormone measures

Gonadal hormone measures were analyzed in a subset of females. Throughout self-administration, vaginal lavage was conducted daily to collect loose vaginal cells. Lavage data were collected for a minimum of 10 consecutive days before, as well as the day of each cue test. Vaginal cytology was conducted to determine the concentration and volume of three main cell types associated with distinct phases of the female estrous cycle: diestrus, as indicated by an abundance of sparse leukocytes, proestrus, as indicated by nucleated epithelial nuclei, and estrus, as indicated by dense, overlapping cornified cells. For the purposes of these experiments, the cycle was split into estrus (proestrus and estrus) and nonestrus (metestrus and diestrus) phases.

To determine the effect of the female estrous cycle on sucrose and/or heroin self-administration, the average number of pellets/infusions earned was calculated and compared across all female rats in estrus versus nonestrus during each day of self-administration. During the early and late abstinence cue tests, the number of active lever presses was averaged and compared for female rats in estrus versus those in nonestrus. Overall locomotion, sniffing, grooming, and hyperactivity were compared for each rat individually and grouped according to whether a given rat was in estrus or nonestrus on the day of the seeking test. Infusions/pellets earned were used as a proxy for opioid/sucrose consumption, whereas active lever presses during the cue test were used to interpret craving.

### Statistical analysis

Three-way ANOVAs were used to compare active lever responses during the day 1 or day 30 cue test with abstinence condition (day 1 vs day 30), sex, and reinforcer (sucrose vs heroin) as between-subjects factors. Three-way ANOVAs were used for overall locomotion, sniffing, grooming, hyperactivity, and 50-kHz USV analyses with sex, reinforcer, and abstinence condition as between-subjects factors. In all cases, if a significant interaction was found after performing ANOVAs, *post hoc* comparisons were made using Bonferroni’s multiple comparisons test.

For comparisons of heroin and sucrose taking across phases of the estrous cycle, mixed model ANOVAs were used to compare infusions/pellets earned during self-administration. Session (self-administration day 1 vs day 2 vs day 3, etc.) was used as the within-subjects factor, and condition (estrus vs nonestrus) was used as the between-subjects factor. Two-way repeated-measures ANOVAs were used to compare active lever presses during the early (day 1) and late (day 30) abstinence cue tests. Abstinence condition (abstinence day 1 vs abstinence day 30) and estrous cycle phase (estrus vs nonestrus) were used as between-subjects factors. Again, if a significant interaction was found after performing ANOVAs, *post hoc* comparisons were made using Sidak’s or Bonferroni’s multiple comparisons test. For comparisons of locomotion, sniffing, grooming, and hyperactivity in estrus versus nonestrus females in early and late abstinence, a two-way ANOVA was used. Abstinence day and estrous cycle phase were both between-subjects factors. Sidak’s and Bonferroni’s multiple comparisons tests were used if an interaction was detected.

## Results

### Incubation of sucrose and heroin craving are associated with reinforcer-specific behavioral profiles

**Figure 1.**
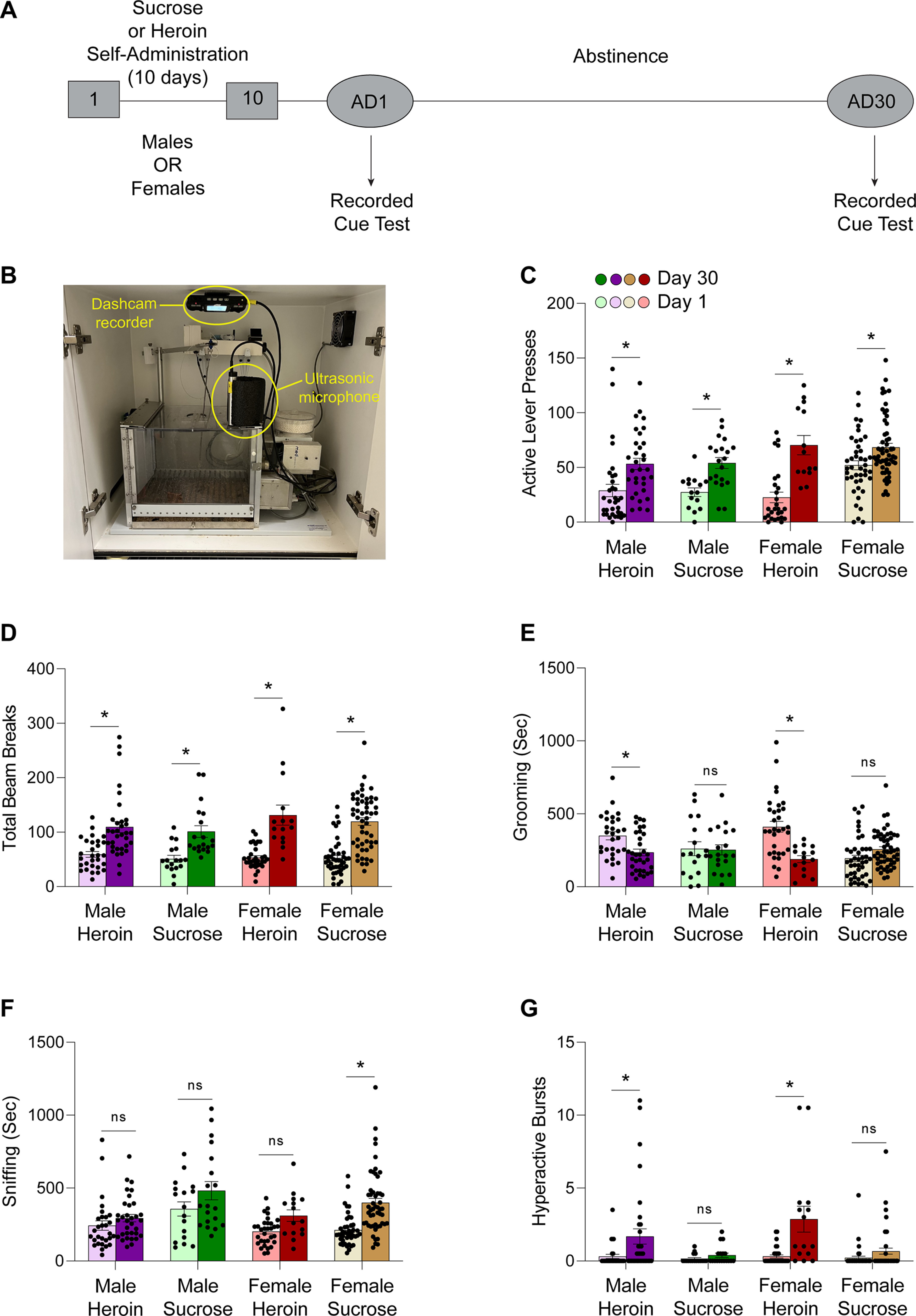
Incubation of craving is associated with sex-specific and reinforcer-specific behavioral signatures. ***A***, Experimental timeline including 10 d of intravenous heroin or oral sucrose self-administration, followed by a recorded cue test after one or 30 d of forced abstinence. ***B***, Cameras were mounted on the ceiling of the sound-attenuating chamber to record movement and behaviors during the cue test. An ultrasonic microphone was positioned flush against the Plexiglas top of the operant box to record USVs. ***C***, Sucrose-exposed and heroin-exposed males and females showed incubation of craving, as indicated by an increase in active levers after 30 d of abstinence compared with 1 d of abstinence. *Post hoc* comparisons revealed that sucrose females responded significantly more than heroin females and sucrose males in early abstinence (day, *F*_(1,232)_ = 52.74, *p *<* *0.0001; day 1 female sucrose vs day 1 female heroin, *p *=* *0.0003; day 1 female sucrose vs day 1 male sucrose, *p *=* *0.0402, Bonferroni’s multiple comparisons test). ***D***, Overall locomotion, as measured by total beam breaks increased in heroin-exposed and sucrose-exposed males and females over 30 d of abstinence (day, *F*_(1,232)_ = 100.6, *p *<* *0.0001). ***E***, Grooming decreased in heroin-exposed rats and was unchanged in sucrose-exposed rats of both sexes after 30 d of abstinence (interaction, *F*_(1,232)_ = 4.318, *p *=* *0.0388; day 1 male heroin vs day 30 male heroin, *p *=* *0.0138; day 1 male sucrose vs day 30 male sucrose, *p *>* *0.9999; day 1 female heroin vs day 30 female heroin, *p *<* *0.0001; day 1 female sucrose vs day 30 female sucrose, *p *=* *0.1848, Bonferroni’s multiple comparisons test). ***F***, Sniffing was unchanged in all conditions, with the exception of an increase in sucrose-exposed females. *Post hoc* comparisons revealed that sucrose males engaged in more sniffing than heroin males in late abstinence, and more sniffing than sucrose females in early abstinence (interaction, *F*_(1,232)_ = 4.304, *p *=* *0.0391; day 1 male heroin vs day 30 male heroin, *p *>* *0.9999; day 1 male sucrose vs day 30 male sucrose, *p *=* *0.1316; day 1 female heroin vs day 30 female heroin, *p* = 0.1848; day 1 female sucrose vs day 30 female sucrose, *p *<* *0.0001, *p *=* *0.1848, Bonferroni’s multiple comparisons test). ***G***, Hyperactivity, as measured by hops and darts across the cage, increased over 30 d of abstinence from heroin, but not sucrose in males and females (interaction, *F*_(1,232)_ = 12.11, *p *=* *0.0006; day 1 male heroin vs day 30 male heroin, *p *=* *0.0050; day 1 male sucrose vs day 30 male sucrose, *p *>* *0.9999; day 1 female heroin vs day 30 female heroin, *p *<* *0.0001; day 1 female sucrose vs day 30 female sucrose, *p *=* *0.7376, Bonferroni’s multiple comparisons test). Additional statistics are listed in Table 1. Data show mean ± SEM; *p < 0.05. AD = abstinence day; ns = not significant, *p* > 0.05.

Male and female rats were allowed to intravenously self-administer (IVSA) heroin (0.10 mg/kg/infusion) or self-administer sucrose pellets for 10 d, and were then assigned to either 1 d (day 1) or 30 d (day 30) of forced abstinence. At the end of each respective abstinence time point, rats were tested for drug-seeking or sucrose-seeking behavior. (Fig. 1*A*). A camera was mounted to the ceiling of the sound-attenuating chamber and an ultrasonic microphone was positioned in the front right corner atop the Plexiglas ceiling of the operant chamber to score behaviors and record USVs, respectively (Fig. 1*B*). Active lever responses increased from abstinence day 1 to abstinence day 30 in heroin-treated and sucrose-treated males and females, indicating incubation of craving (Fig. 1*C*; additional statistics in Table 1; day, *F*_(1,232)_ = 52.74, *p *<* *0.0001). Overall locomotion, as measured by the total number of times a rat crossed one of the superimposed beams bisecting the operant chamber in the video recording, increased over 30 d of abstinence from both heroin and sucrose in both sexes (Fig. 1*D*; additional statistics in Table 1; day, *F*_(1,232)_ = 100.6, *p *<* *0.0001). Grooming decreased in heroin males and females, and was unchanged in sucrose males and females after 30 d of abstinence (Fig. 1*E*; additional statistics in Table 1; interaction, *F*_(1,232)_ = 4.318, *p *=* *0.0388; day 1 male heroin vs day 30 male heroin, *p *=* *0.0138; day 1 male sucrose vs day 30 male sucrose, *p *>* *0.9999; day 1 female heroin vs day 30 female heroin, *p *<* *0.0001; day 1 female sucrose vs day 30 female sucrose, *p *=* *0.1848, Bonferroni’s multiple comparisons test). Sniffing was unchanged in all conditions, with the exception of an increase in sucrose-exposed females (Fig. 1*F*; additional statistics in Table 1; interaction, *F*_(1,232)_ = 4.304, *p *=* *0.0391; day 1 male heroin vs day 30 male heroin, *p *>* *0.9999; day 1 male sucrose vs day 30 male sucrose, *p *= 0.1316; day 1 female heroin vs day 30 female heroin, *p *=* *0.1848; day 1 female sucrose vs day 30 female sucrose, *p *<* *0.0001, Bonferroni’s multiple comparisons test). Hyperactivity, as measured by hops and darts across the cage, increased over 30 d of abstinence from heroin, but not sucrose in males and females (Fig. 1*G*; additional statistics in Table 1; interaction, *F*_(1,232)_ = 12.11, *p *=* *0.0006; day 1 male heroin vs day 30 male heroin, *p *=* *0.0050; day 1 male sucrose vs day 30 male sucrose, *p *>* *0.9999; day 1 female heroin vs day 30 female heroin, *p *<* *0.0001; day 1 female sucrose vs day 30 female sucrose, *p *=* *0.7376, Bonferroni’s multiple comparisons test).

**Table 1:** Additional statistics associated with results from **Figures 1, 2**

Figure	Factors	*F* values	*p*-values
Fig. 1*C*	Day	*F*_(1,232)_ = 52.74	*p* < 0.0001
	Sex	*F*_(1,232)_ = 9.825	*p* = 0.0019
	Reinforcer	*F*_(1,232)_ = 2.824	*p* = 0.0942
	Day × sex	*F*_(1,232)_ = 0.6877	*p* = 0.4078
	Day × reinforcer	*F*_(1,232)_ = 3.340	*p* = 0.0689
	Sex × reinforcer	*F*_(1,232)_ = 3.139	*p* = 0.0777
	Day × sex × reinforcer	*F*_(1,232)_ = 4.549	*p* = 0.0340
Fig. 1*D*	Day	*F*_(1,232)_ = 100.6	*p* < 0.0001
	Sex	*F*_(1,232)_ = 2.124	*p* = 0.1464
	Reinforcer	*F*_(1,232)_ = 1.397	*p* = 0.2384
	Day × sex	*F*_(1,232)_ = 3.380	*p* = 0.0673
	Day × reinforcer	*F*_(1,232)_ = 0.2113	*p* = 0.6461
	Sex × reinforcer	*F*_(1,232)_ = 0.02831	*p* = 0.8665
	Day × sex × reinforcer	*F*_(1,232)_ = 0.2019	*p* = 0.6536
Fig. 1*E*	Day	*F*_(1,232)_ = 10.78	*p* = 0.0012
	Sex	*F*_(1,232)_ = 0.2969	*p* = 0.5864
	Reinforcer	*F*_(1,232)_ = 6.620	*p* = 0.0107
	Day × sex	*F*_(1,232)_ = 0.1863	*p* = 0.6664
	Day × reinforcer	*F*_(1,232)_ = 20.91	*p* < 0.0001
	Sex × reinforcer	*F*_(1,232)_ = 0.8497	*p* = 0.3576
	Day × sex × reinforcer	*F*_(1,232)_ = 4.318	*p* = 0.0388
Fig. 1*F*	Day	*F*_(1,232)_ = 23.20	*p* < 0.0001
	Sex	*F*_(1,232)_ = 6.569	*p* = 0.0110
	Reinforcer	*F*_(1,232)_ = 16.82	*p* < 0.0001
	Day × sex	*F*_(1,232)_ = 1.523	*p* = 0.2184
	Day × reinforcer	*F*_(1,232)_ = 2.408	*p* = 0.1221
	Sex × reinforcer	*F*_(1,232)_ = 4.304	*p* = 0.0391
	Day × sex × reinforcer	*F*_(1,232)_ = 0.001682	*p* = 0.9673
Fig. 1*G*	Day	*F*_(1,231)_ = 24.49	*p* < 0.0001
	Sex	*F*_(1,231)_ = 2.717	*p* = 0.1007
	Reinforcer	*F*_(1,231)_ = 16.05	*p* < 0.0001
	Day × sex	*F*_(1,231)_ = 2.268	*p* = 0.1334
	Day × reinforcer	*F*_(1,231)_ = 12.11	*p* = 0.0006
	Sex × reinforcer	*F*_(1,231)_ = 0.8109	*p* = 0.3688
	Day × sex × reinforcer	*F*_(1,231)_ = 1.068	*p* = 0.3025
Fig. 2	Day	*F*_(1,51)_ = 65.73	*p* < 0.0001
	Sex	*F*_(1,51)_ = 84.83	*p* < 0.0001
	Reinforcer	*F*_(1,51)_ = 69.82	*p* < 0.0001
	Day × sex	*F*_(1,51)_ = 67.04	*p* < 0.0001
	Day × reinforcer	*F*_(1,51)_ = 56.88	*p* < 0.0001
	Sex × reinforcer	*F*_(1,51)_ = 68.35	*p* < 0.0001
	Day × sex × reinforcer	*F*_(1,51)_ = 60.12	*p* < 0.0001

### Heroin-exposed male rats show an increase in 50-kHz USVs after 30 d of forced abstinence

**Figure 2.**
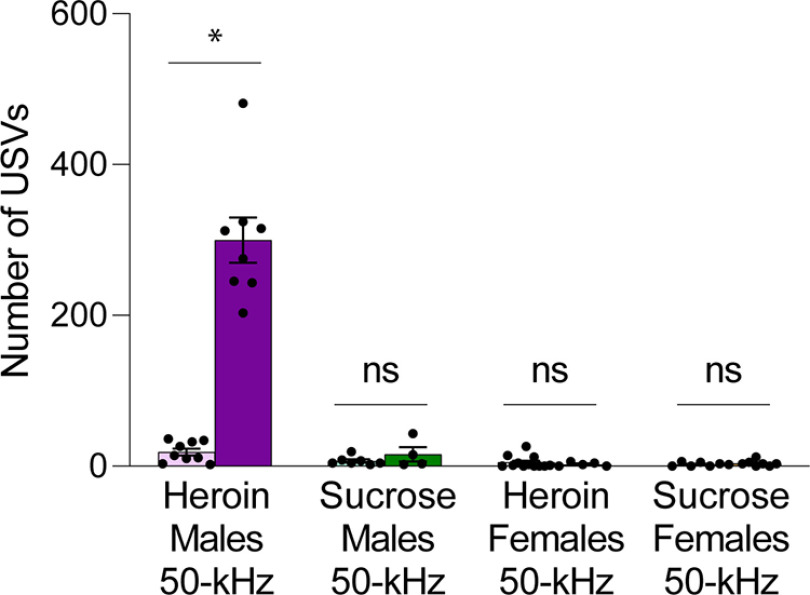
Incubation of heroin craving is associated with an increase in 50-kHz USVs in males only (interaction, *F*_(1,51)_ = 60.12, *p *<* *0.0001; day 1 heroin male 50-kHz calls vs day 30 heroin male 50-kHz calls, *p *<* *0.0001; day 1 sucrose male 50-kHz calls vs day 30 sucrose male 50-kHz calls, *p *>* *0.9999; day 1 heroin female 50-kHz calls vs day 30 heroin female 50-kHz calls, *p *>* *0.9999; day 1 sucrose female 50-kHz calls vs day 30 sucrose female 50-kHz calls, *p *> 0.9999, Bonferroni’s multiple comparisons test). Additional statistics are listed in Table 1. Data show mean ± SEM; *p < 0.05.

22-kHz USVs are unchanged in heroin-exposed males and females (data not shown); 50-kHz USVs increased only in heroin-exposed male rats and were unchanged in all other groups after 30 d of abstinence (Fig. 2; additional statistics in Table 1; interaction, *F*_(1,51)_ = 60.12, *p *< 0.0001; day 1 heroin male 50-kHz calls vs day 30 heroin male 50-kHz calls, *p *<* *0.0001; day 1 sucrose male 50-kHz calls vs day 30 sucrose male 50-kHz calls, *p *> 0.9999; day 1 heroin female 50-kHz calls vs day 30 heroin female 50-kHz calls, *p *>* *0.9999; day 1 sucrose female 50-kHz calls vs day 30 sucrose female 50-kHz calls, *p *>* *0.9999, Bonferroni’s multiple comparisons test). Very few 22-kHz calls were observed during any of the cue tests. Many were short in length and captured in conjunction with a series of 50-kHz calls. Compared with 50-kHz calls, 22-kHz calls were very infrequent and did not change from early to late abstinence in either sex for either reinforcer.

### Estrous cycle does not impact heroin or sucrose self-administration nor incubation of craving

**Figure 3.**
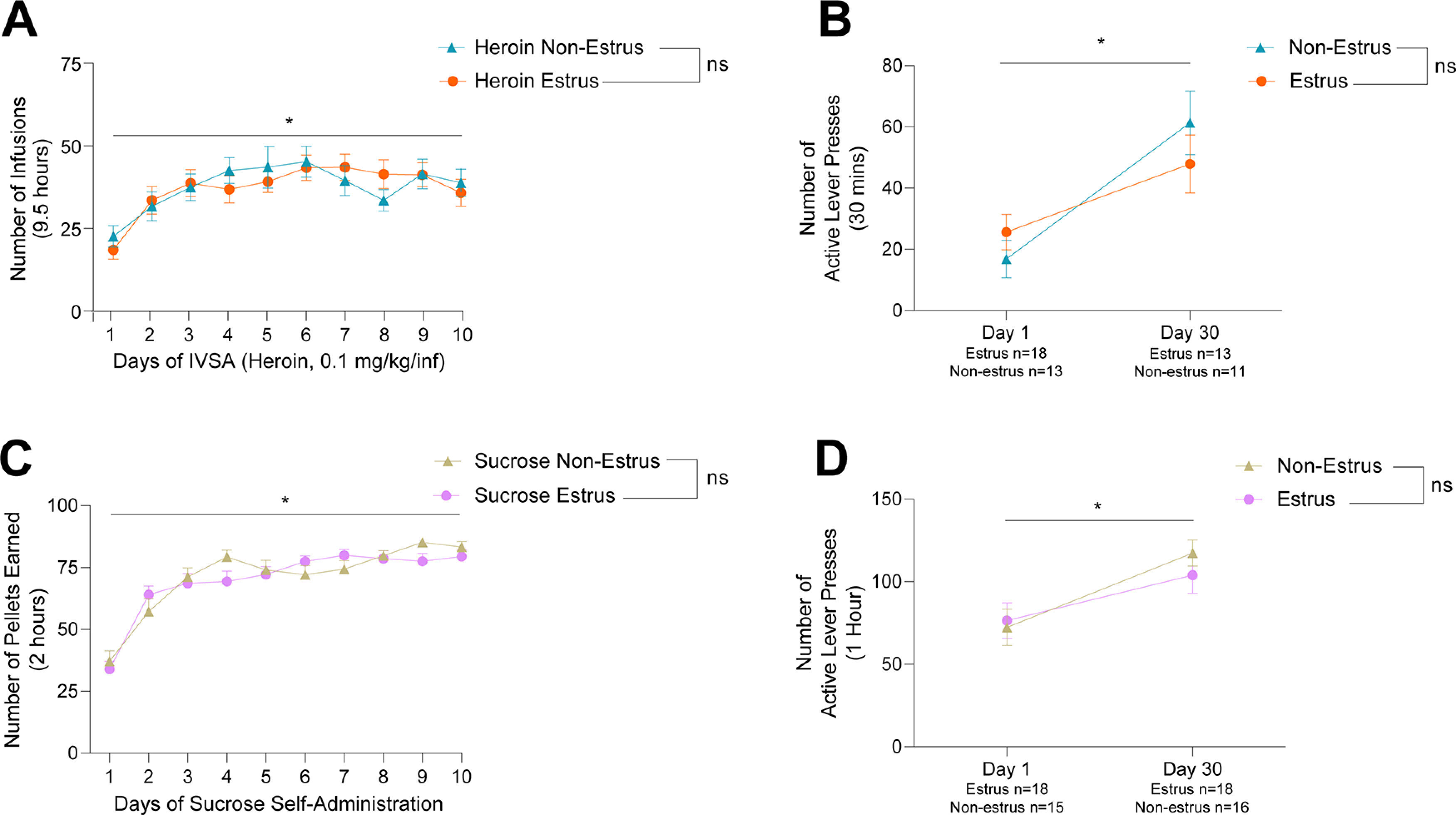
The effect of the female estrous cycle phase (estrus vs nonestrus) on heroin and sucrose self-administration and craving. ***A***, Female heroin-treated rats earned more infusions over 10 d of intravenous self-administration regardless of estrous cycle phase (time, *F*_(9,541)_ = 3.821, *p *=* *0.0001; estrus, *F*_(3,148)_ = 0.5299, *p *=* *0.6624; interaction, *F*_(27,541)_ = 4.405, *p *<* *0.0001). ***B***, Females demonstrated incubation of heroin craving, as indicated by an increase in active lever responses during a 30-min cue test following 30 d of abstinence, and there were no differences in active lever responses between estrus and nonestrus females at either abstinence time point (time, *F*_(1,51)_ = 18.02, *p *<* *0.0001; estrus, *F*_(1,51)_ = 0.0881, *p *=* *0.7677; interaction, *F*_(1,51)_ = 1.989, *p *=* *0.1645). ***C***, Female sucrose-treated rats earned more sucrose pellets over 10 d of oral self-administration regardless of estrous cycle phase (time, *F*_(9,760)_ = 66.68, *p *<* *0.0001; estrus, *F*_(1,201)_ = 0.2342, *p *=* *0.6290; interaction, *F*_(9,760)_ = 0.8406, *p *=* *0.5787). ***D***, Females demonstrated incubation of sucrose craving, as indicated by an increase in active lever responses during a 1-h cue test following 30 d of abstinence, and there were no differences in active lever responses between estrus and nonestrus females at either abstinence time point (time, *F*_(1,63)_ = 12.75, *p *=* *0.0007; estrus, *F*_(1,63)_ = 0.2057, *p *=* *0.6517; interaction, *F*_(1,63)_ = 0.7248, *p *=* *0.6517). Data show mean ± SEM; *p < 0.05.

Over 10 d of self-administration females took increasing amounts of heroin (Fig. 3*A*; time, *F*_(9,541)_ = 3.821, *p *=* *0.0001; interaction, *F*_(27,541)_ = 4.405, *p *<* *0.0001). There was no difference in heroin taking between females in estrus versus those in nonestrus (Fig. 3*A*; estrus, *F*_(3,148)_ = 0.5299, *p *=* *0.6624). Female rats experienced incubation of heroin craving following 30 d of abstinence (Fig. 3*B*; time, *F*_(1,51)_ = 18.02, *p *<* *0.0001); however, craving was not affected by phase of the estrous cycle (Fig. 3*B*; estrus, *F*_(1,51)_ = 0.0881, *p *=* *0.7677; interaction, *F*_(1,51)_ = 1.989, *p *=* *0.1645). Similarly, increasing amounts of sucrose were taken over 10 d of self-administration (Fig. 3*C*; time, *F*_(9,760)_ = 66.68, *p *<* *0.0001), and there were no differences between females in estrus versus those in nonestrus (Fig. 3*C*; estrus, *F*_(1,201)_ = 0.2342, *p *=* *0.6290; interaction, *F*_(9,760)_ = 0.8406, *p *=* *0.5787). Additionally, females experienced incubation of craving for the natural reward sucrose (Fig. 3*D*; time, *F*_(1,63)_ = 12.57, *p *=* *0.0007), and there was no effect of estrous cycle phase on sucrose craving (Fig. 3*D*; estrus, *F*_(1,63)_ = 0.2057, *p *=* *0.6517; interaction, *F*_(1,63)_ = 0.7248, *p *=* *0.6517).

### Locomotion is the only behavior enhanced during estrus in females after 30 d of abstinence from heroin compared with nonestrus

**Figure 4.**
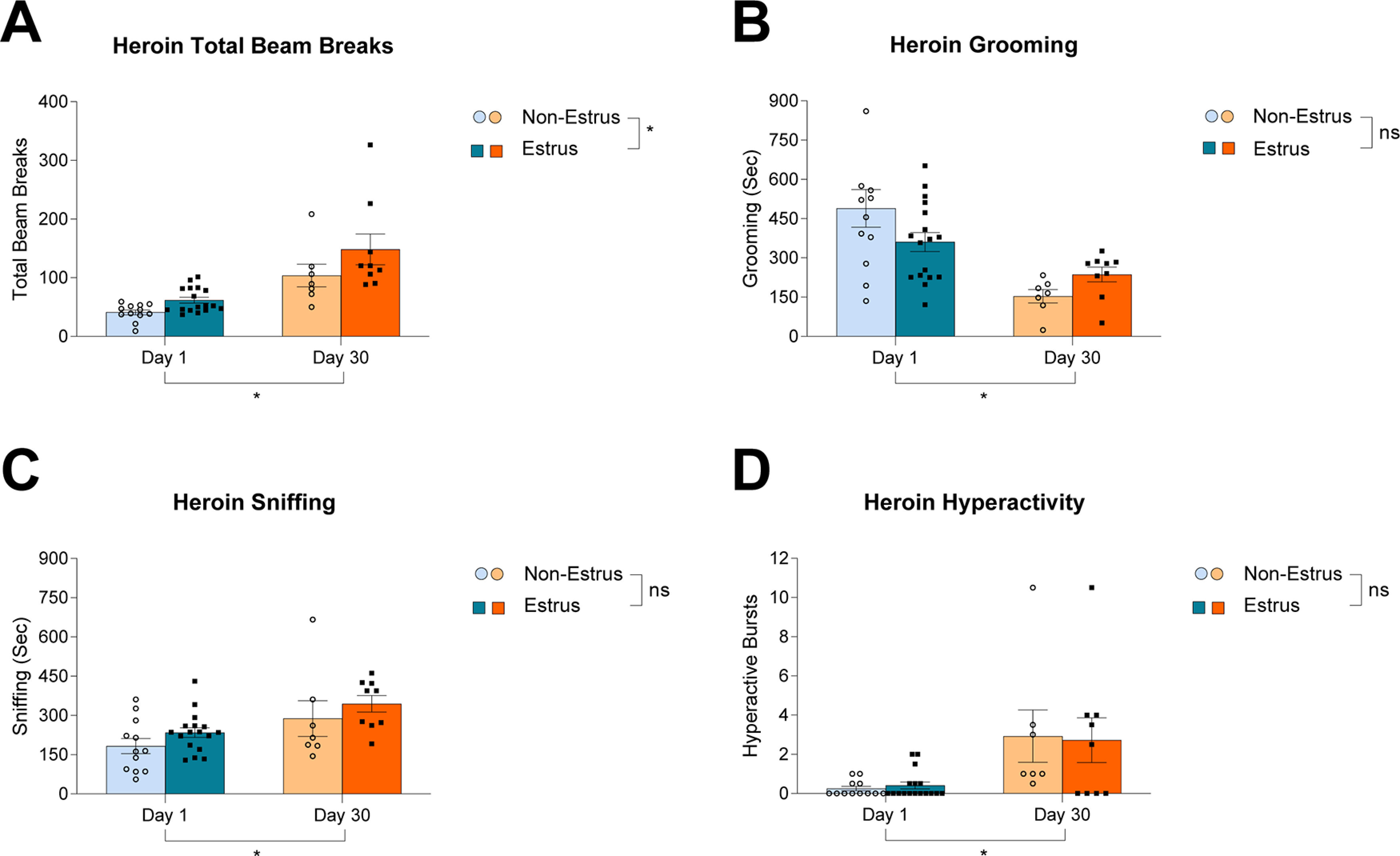
The effect of the female estrous cycle phase (estrus vs nonestrus) on addiction-related behaviors following one or 30 d of abstinence from heroin. ***A***, Total beam breaks increased over 30 d of abstinence, and were enhanced in estrus females compared with nonestrus females (time, *F*_(1,45)_ = 33.58, *p *<* *0.0001; estrus, *F*_(1,45)_ = 6.151, *p *=* *0.0169; interaction, *F*_(1,45)_ = 0.5535, *p *=* *0.4608). ***B***, Grooming decreased over 30 d of abstinence regardless of estrous cycle phase (time, *F*_(1,45)_ = 18.30, *p *=* *0.0001; estrus, *F*_(1,45)_ = 0.0093, *p *=* *0.9235; interaction, *F*_(1,45)_ = 1.762, *p *=* *0.1911). ***C***, Sniffing increased over 30 d of abstinence regardless of estrous cycle phase (time, *F*_(1,45)_ = 9.245, *p *=* *0.0039; estrus, *F*_(1,45)_ = 1.018, *p *=* *0.3183; interaction, *F*_(1,45)_ = 0.0221, *p *=* *0.8826). ***D***, Hyperactivity increased over 30 d of abstinence and there was no effect of estrous cycle phase (time, *F*_(1,45)_ = 15.36, *p *=* *0.0003; estrus, *F*_(1,45)_ = 0.0558, *p *=* *0.8144; interaction, *F*_(1,45)_ = 0.2112, *p *=* *0.6481). Data show mean ± SEM; *p < 0.05.

Total beams breaks increased from day 1 to day 30 of abstinence in heroin-treated females (Fig. 4*A*; time, *F*_(1,45)_ = 33.58, *p *<* *0.0001). Estrus females showed higher locomotion than nonestrus females (Fig. 4*A*; estrus, *F*_(1,45)_ = 6.151, *p *=* *0.0169; interaction, *F*_(1,45)_ = 0.5535, *p *=* *0.4608). Heroin females experienced a decrease in grooming over 30 d of abstinence that was not affected by phase of the estrous cycle (Fig. 4*B*; time, *F*_(1,45)_ = 18.30, *p *=* *0.0001; estrus, *F*_(1,45)_ = 0.0093, *p *=* *0.9235; interaction, *F*_(1,45)_ = 1.762, *p *=* *0.1911). Sniffing increased over 30 d of abstinence from heroin regardless of phase of the estrous cycle (Fig. 4*C*; time, *F*_(1,45)_ = 9.245, *p *=* *0.0039; estrus, *F*_(1,45)_ = 1.018, *p *=* *0.3183; interaction, *F*_(1,45)_ = 0.0221, *p *=* *0.8826). Interestingly, the increase in hyperactivity over 30 d of abstinence from heroin was not affected by the phase of the estrous cycle (Fig. 4*D*; time, *F*_(1,45)_ = 15.36, *p *=* *0.0003; estrus, *F*_(1,45)_ = 0.0558, *p *=* *0.8144; interaction, *F*_(1,45)_ = 0.2112, *p *=* *0.6481).

### Locomotion and hyperactivity are enhanced in estrus females after 30 d of abstinence from sucrose

**Figure 5.**
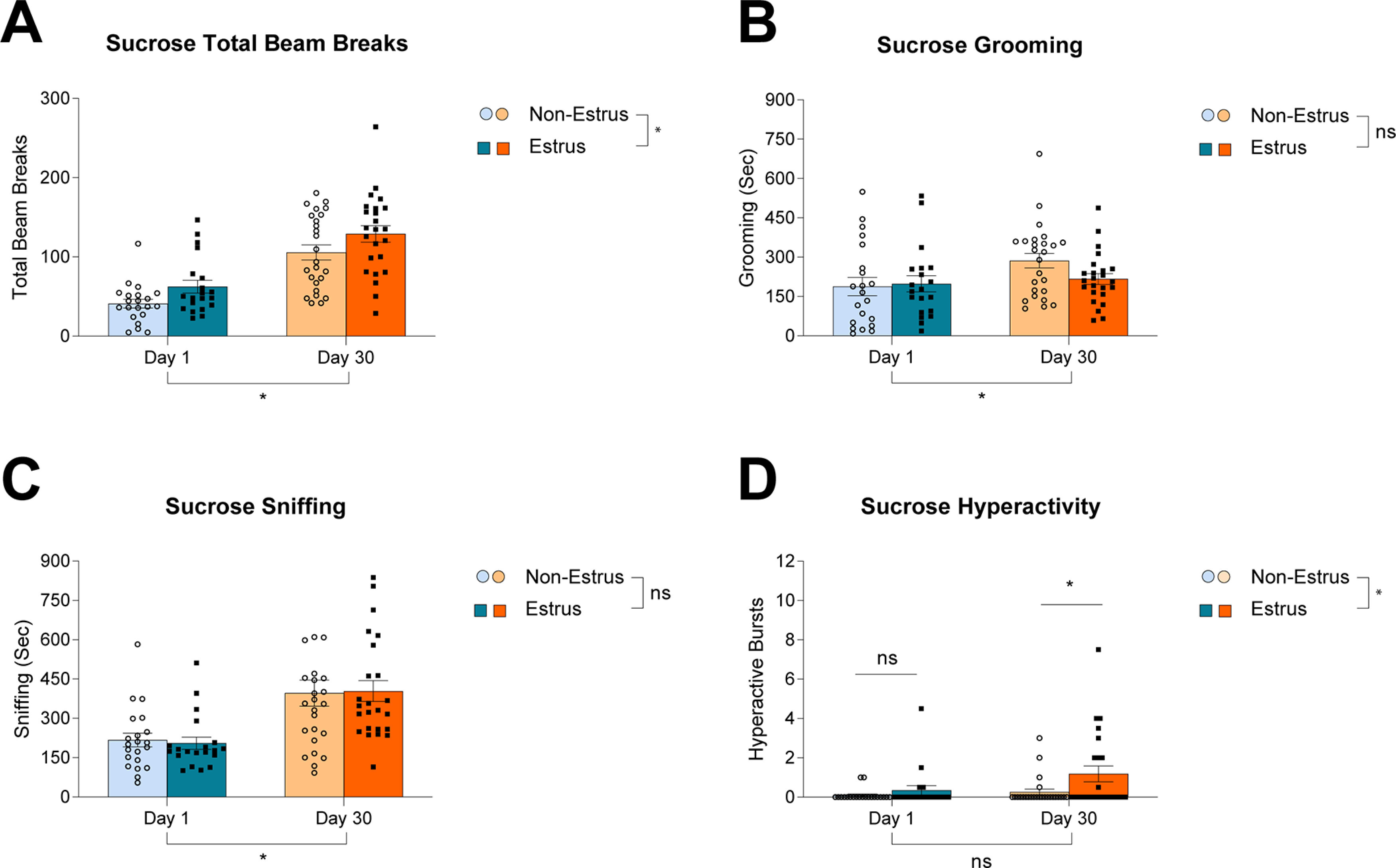
The effect of the female estrous cycle phase (estrus vs nonestrus) on addiction-related behaviors following one or 30 d of abstinence from sucrose. ***A***, Total beam breaks increased over 30 d of abstinence, and were enhanced in estrus females compared with nonestrus females (time, *F*_(1,86)_ = 54.91, *p *<* *0.0001; estrus, *F*_(1,86)_ = 6.386, *p *=* *0.0133; interaction, *F*_(1,86)_ = 0.0115, *p *=* *0.9150). ***B***, Grooming increased over 30 d of abstinence regardless of estrous cycle phase (time, *F*_(1,86)_ = 4.194, *p *=* *0.0436; estrus, *F*_(1,86)_ = 1.091, *p *=* *0.2993; interaction, *F*_(1,86)_ = 1.974, *p *=* *0.11 636). ***C***, Sniffing increased over 30 d of abstinence regardless of estrous cycle phase (time, *F*_(1,86)_ = 23.98, *p *<* *0.0001; estrus, *F*_(1,86)_ = 0.003, *p *=* *0.9568; interaction, *F*_(1,86)_ = 0.064, *p *=* *0.8006). ***D***, Hyperactivity was enhanced at day 30 in estrus females, and there was no effect of estrous cycle on hyperactivity at day 1 (time, *F*_(1,86)_ = 3.923, *p *=* *0.0508; estrus, *F*_(1,86)_ = 5.459, *p *=* *0.0218; interaction, *F*_(1,86)_ = 1.767, *p *=* *0.1872; day 1 estrus vs nonestrus, *p *>* *0.9999; day 30 estrus vs nonestrus, *p *=* *0.0479, Bonferroni’s multiple comparisons test). Data show mean ± SEM; *p < 0.05.

As with heroin-treated females, overall locomotion, as measured by total beam breaks increased over 30 d of abstinence from sucrose (Fig. 5*A*; time, *F*_(1,86)_ = 54.91, *p *<* *0.0001). Again, locomotion was enhanced in estrus females compared with nonestrus females (Fig. 5*A*; estrus, *F*_(1,86)_ = 6.386, *p *=* *0.0133; interaction, *F*_(1,86)_ = 0.0115, *p *=* *0.9150). Grooming increased over 30 d of abstinence from sucrose (Fig. 5*B*; time *F*_(1,86)_ = 4.194, *p *=* *0.0436), but was not affected by phase of the estrous cycle (Fig. 5*B*; estrus, *F*_(1,86)_ = 1.091, *p *=* *0.2993; interaction, *F*_(1,86)_ = 1.974, *p *=* *0.11 636). Similarly, sniffing increased over 30 d of abstinence from sucrose (Fig. 5*C*; time, *F*_(1,86)_ = 23.98, *p *<* *0.0001), but was not affected by phase of the estrous cycle (Fig. 5*C*; estrus, *F*_(1,86)_ = 0.003, *p *=* *0.9568; interaction, *F*_(1,86)_ = 0.064, *p *=* *0.8006). Hyperactivity was enhanced in estrus females at day 30 of abstinence, but was not affected by estrous cycle phase in early abstinence (Fig. 5*D*; time, *F*_(1,86)_ = 3.923, *p *=* *0.0508; estrus, *F*_(1,86)_ = 5.459, *p *=* *0.0218; interaction, *F*_(1,86)_ = 1.767, *p *=* *0.1872; day 1 estrus vs nonestrus, *p *>* *0.9999; day 30 estrus vs nonestrus, *p *=* *0.0479, Bonferroni’s multiple comparisons test).

## Discussion

This study aimed to refine a more robust picture of behavioral and affective states that accompany opioid seeking after extended abstinence, when craving is high. After chronic self-administration of either intravenous heroin or the natural reward sucrose, rats underwent an abstinence paradigm that is known to induce craving after 30 d (Lu et al., 2004). Sucrose is a natural reward that acts on many of the same brain regions as drugs of abuse and leads to enhanced craving. Thus, it was important to establish behaviors that change in response to extended abstinence and cue re-exposure generally, and subsequently to differentiate opioid-specific behavioral changes. To that end, we explored overall locomotion, sniffing, grooming, hyperactivity, USVs, and estrous cycle phase. Results indicate a reinforcer-specific behavioral profile that emerges after 30 d of forced abstinence in heroin-exposed rats. Interestingly, total beam breaks increased over 30 d of abstinence regardless of sex and reinforcer, indicating that increased craving is associated with a general increase in locomotion. Heroin exposure resulted in a unique behavioral signature, including a decrease in grooming and an increase in hyperactivity that was seen in both sexes but did not generalize to the natural reward sucrose. Sniffing showed a sex-specific and reinforcer-specific effect, such that it increased in sucrose-treated females over 30 d of abstinence, and was unchanged in all other groups. Heroin-exposed males made significantly more 50-kHz USVs during the late abstinence seeking test when craving was high, which was not seen in females, nor sucrose-exposed rats of either sex. Estrous cycle phase did not influence self-administration nor craving for either reinforcer. However, locomotion and/or hyperactivity were enhanced in estrous females in a reinforcer-specific manner. These results underscore the notion that opioids cause long-lasting neural adaptations, which take effect and persist throughout abstinence to prime the system for increased craving and compulsive drug seeking when re-exposed to drug-paired cues. These changes are characterized by decreased grooming and increased hyperactivity, which represent an opioid-specific behavioral profile of craving in response to cue re-exposure. An increase in positive-valence affective state is thought to drive this drug-seeking behavioral signature, although this may be sex-specific. The mechanism for explaining the different behavioral profiles in heroin-exposed versus sucrose-exposed rats is undetermined and outside the scope of this report. Ultimately, the goal is that these descriptive data lay the foundation for further exploration of such a mechanism. Taken together, these results are promising in terms of developing treatments tailored to opioid craving and associated behaviors without interrupting the natural reward system.

### Increase in lever pressing is associated with increased locomotion in males and females, regardless of reinforcer

Re-exposure to reward-paired cues not only induces an increase in seeking behavior, but also a general increase in overall locomotion, as measured by beam breaks. There are similarities between changes in locomotion/stereotypies and incubation of craving, such that both persist after long periods of abstinence (Paulson et al., 1991; Pickens et al., 2011; Madangopal et al., 2019). For example, locomotion increases during a cue-induced reinstatement test following chronic sucrose self-administration and 30 d of abstinence (Grimm et al., 2007; Aoyama et al., 2014; Harkness et al., 2016), although in some of these studies, rats had undergone extinction training before reinstatement. Following exposure to experimenter-delivered morphine, a priming injection stimulated increased locomotor sensitization after three and nine weeks of abstinence (Vanderschuren et al., 1997). Others have shown that this persists for up to three months of abstinence (Powell and Holtzman, 2001). Although these studies (and the bulk of studies examining sensitization) focus on changes with opioids on board, one report found that overall locomotion is depressed 24 h into abstinence without re-exposure to heroin. Although it is unclear whether this change reached significance, locomotion appears to increase following 14 d of abstinence, again without a priming injection (Leri et al., 2003). All of these aforementioned conclusions were based on male subjects only. This study corroborates these findings and extends them to females.

Locomotion and reinstatement are mediated by overlapping neurotransmitter mechanisms in the ventral tegmental area and accumbens, whereby activation of glutamate receptors on accumbal dopamine terminals leads to the increase in dopamine that drives increased locomotion and motivation (Wu et al., 1993; Wise, 2004). In fact, reducing locomotion and seeking sometimes cannot be dissociated. For example, dopamine D1 receptor antagonist SCH 23390 attenuates overall locomotion and sucrose seeking, which are increased after 30 d of abstinence without extinction training (Grimm et al., 2011). Additionally, treatments that reduce incubation of cocaine craving also reduce overall locomotion after 30+ d of forced abstinence (Murray et al., 2021). Thus, there is an overlapping system that contributes to behavioral sensitization with drugs on board, cue-induced relapse-like behavior, and locomotion. Here, we show that these drug-induced neural adaptations can persist and be triggered by cue re-exposure even after prolonged abstinence.

### In both sexes, chronic heroin exposure and extended abstinence results in an opioid-specific behavioral profile

Although craving and overall locomotion increase over 30 d of abstinence regardless of sex and reinforcer, other behaviors, like grooming and hyperactivity change uniquely following exposure to heroin versus sucrose. Most of the research on incubation of craving has used responses on a previously reward-paired lever to assess craving, regardless of reinforcer. Here, we show that other behaviors, such as grooming and hyperactivity, may be better suited for quantifying *opioid* craving. One important limitation of this study is that certain self-administration parameters (session duration and continuous vs intermittent access) were inconsistent across reinforcers, which possibly contributes to the behavioral differences seen here. Although a mechanism for driving these behavioral changes is undetermined, these unique behavioral profiles are significant and relevant because they (1) underscore the wealth of data indicating that increased active lever presses occur in males and females on cue re-exposure after prolonged abstinence, while (2) adding more nuanced and robust measurements of opioid craving specifically. These data can help improve preclinical models of craving/relapse-like behavior while also serving as a powerful foundation for additional mechanistic exploration.

The decrease in grooming and increase in hyperactivity that are associated with peak heroin craving may represent a hyperactive or compulsive behavioral profile similar to compulsive drug seeking. Noradrenergic hyperactivity can lead to hyperarousal, trouble focusing, and overactivity (Yamamoto et al., 2014). Treatments that reduce noradrenergic outflow have been shown to reduce cue-induced opioid craving (Sinha et al., 2007). Thus, one interpretation is that cue-induced heroin craving in late abstinence is reflective of a hyperactive state, a preoccupation with drug seeking, and a decrease in self-directed behaviors, such as grooming. Grooming and hyperactivity are associated with cocaine-induced and opioid-induced behavioral sensitization (Becker et al., 2001; Zhan et al., 2015), although not many studies have examined them in the context of cue-induced craving without re-exposure to the drug itself. This study lays the groundwork for further exploration of potential mechanisms underlying the behavioral profile associated with opioid-paired cue re-exposure.

It is interesting that many of the behaviors – incubation of craving, increased locomotion, and the heroin-specific decrease in grooming, and increase in hyperactivity– were consistently altered in both sexes. The only recorded behavior that was differentially affected in males and females was the sucrose-specific increase in sniffing seen in females, but not males. Notably, although sucrose-exposed females respond more than sucrose-exposed males during early abstinence, there were no observed sex differences in heroin seeking at either time point. This is consistent with other reports that males and females experience similar increases in opioid-seeking behavior after extended abstinence (Venniro et al., 2019). Compared with males, females show enhanced incentive sensitization to cocaine following intermittent self-administration (Kawa and Robinson, 2019), as well as behavioral sensitization to experimenter-delivered amphetamine (Van Haaren and Meyer, 1991). In some cases, behaviors like grooming are uniquely affected in only females and unchanged in males when morphine is on board (Zhan et al., 2015). Here, the magnitude of change in stereotypies and locomotion are similar in males and females, with the exception of sniffing in sucrose-exposed females. A possible explanation is that males and females react differently when opioids are present, but that over time, the opioid-induced and cue-induced behavioral changes become more comparable across sexes. This would help explain why males and females show a similar increase in cue-induced opioid craving over 30 d of abstinence (Venniro et al., 2019). Overall, this is promising in terms of designing treatments administered throughout abstinence that are likely to be effective in both sexes at reducing craving and other behaviors associated with compulsive drug seeking.

### Extended abstinence from heroin is associated with an increase in 50-kHz USVs in males, but not females

Another sex-specific change was reflected in 50-kHz USVs. USVs were recorded during seeking tests to determine whether there is an affective component of drug/sucrose seeking in early versus late abstinence, when craving is high and other heroin-induced behavioral changes emerge. Audio recording of craving tests on day 1 and day 30 of abstinence revealed no change in 22-kHz USVs in either sex, and an increase in 50-kHz USVs in males, but not females. 22- and 50-kHz USVs were unchanged in sucrose-exposed males and females. The lack of 22-kHz USVs is inconsistent with previous reports that when a conditioned cue is no longer predictive of a reward, rats will emit 22-kHz calls (Coffey et al., 2013). Here, there were few 22-kHz calls during any of the cue tests for either sex/reinforcer. The lack of 22-kHz USVs is consistent with the idea that somatic withdrawal symptoms are not driving craving behavior. Although rats may experience withdrawal-like behavior in early abstinence following chronic opioid self-administration (Gipson et al., 2021; Seaman and Collins, 2021), withdrawal symptoms subside within the first few days of abstinence (Welsch et al., 2020). Yet, craving continues to intensify for several weeks. As such, withdrawal signs and craving follow opposite trajectories and one is unlikely to affect the other. Interestingly, protracted forced abstinence from cocaine (30 or 60 d) does not lead to a difference in 22- nor 50-kHz calls during a contextual reinstatement test compared with calls made during self-administration (Barker et al., 2014), although in this study, USVs were not collected during an early abstinence seeking test. Here, 50-kHz, but not 22-kHz, USVs change across abstinence in a sex-specific and reinforcer-specific manner.

Preclinical studies reveal that there are relatively fewer sex differences associated with opioid-related behaviors, including relapse (Nicolas et al., 2021). Here, males and females show similar patterns of relapse-like behavior, and yet females do not display an increase in 50-kHz USVs during the late abstinence relapse test when craving is high. It is likely that males emit 50-kHz USVs as an anticipatory behavior during re-exposure to opioid-paired cues. This is consistent with studies demonstrating that males report elevated moods before and during cocaine and alcohol relapse events (McKay et al., 1996; Rubin et al., 1996). Other studies also interpret 50-kHz USVs as positive-valence affective states in anticipation of social and drug rewards (Barfield et al., 1979; Ma et al., 2010). In fact, cues predictive of electrical stimulation of reward-related brain regions elicit increased 50-kHz calls in males (Burgdorf et al., 2000). Conversely, women are more prone to anxiety-driven, stress-driven, and depression-driven cocaine relapse (for review, see McKay et al., 1996; Rubin et al., 1996; Becker and Koob, 2016). Women report a greater increase in sadness and decrease in joy in response to opioid-paired imagery and paraphernalia compared with men (Yu et al., 2007). As with many observed sex differences, further research is needed to delineate why other behavioral end points are similar at abstinence day 30, and yet there is a sex difference in 50-kHz USVs. In other studies examining USVs, females are often not included or not compared with males directly. One possibility is that females are more prone to stress-induced relapse. Re-exposure to drug-paired cues in the absence of drug delivery is likely to evoke mild stress (Schultz et al., 1997), and stress-induced reinstatement does increase over time (Sinha et al., 2011). However, it can be argued that the cue test is not as stressful as the stimuli used to precipitate stress-induced seeking, such as a foot shock. Therefore, it is likely not sufficient to induce 22-kHz calls nor 50-kHz calls in females, which would be consistent with a positive affect-induced relapse event. To that point, if a stress-primed reinstatement model was used in place of the cue-induced model here, females may show a greater number of USVs as they are potentially more sensitive to stress-induced relapse.

In males, the increase in 50-kHz positive-valence affective calls, increase in locomotion, and increase in hyperactivity that accompany increased drug seeking may reflect an overactive or “manic”-like state. The simultaneous decrease in grooming may reflect a decrease in self-directed behaviors that is generally consistent with a compulsive drug seeking profile. This should be interpreted with caution so as not to over-anthropomorphize these measures. The intention is simply to characterize the behavioral profiles in a way that may lead to further exploration of underlying mechanisms. For example, these changes are potentially caused by dopamine D1 receptor activation in the nucleus accumbens shell, given that dopamine D1 receptors are linked to initiating anticipatory 50-kHz calls (Simola and Brudzynski, 2018), and D1 receptor antagonism reduces these calls (Buck et al., 2014). Of course, the dopamine system is also extensively linked to drug reward (Wise and Bozarth, 1985), motivation to seek rewards (for review, see Volkow et al., 2017), as well as pathological manic states when there is an excess of dopamine (Ashok et al., 2017). Moreover, methamphetamine-induced 50-kHz calls are attenuated by anti-mania drugs and have, in the past, been used to quantify mania-like behavior in methamphetamine-treated rats (Wöhr, 2021). Additional studies are needed to ascertain the exact underlying mechanisms that connect increased locomotion, hyperactivity, and 50-kHz calls to decreased grooming and increased heroin seeking; however, their coexistence could represent a state of hyperarousal and compulsive drug seeking possibly mediated by the dopamine system.

### Females take increasing amounts of heroin and sucrose, and experience incubation of craving regardless of estrous cycle phase

Overall, this study shows few sex differences in terms of opioid-related behaviors. It is possible that sex differences will emerge when females are tested in a specific phase of the estrous cycle, and this effect may be obscured given the large sample sizes and estrous cycle variability across samples. Although there are many published reports indicating that the female estrous cycle impacts stimulant-related and nicotine-related behaviors (Lynch, 2008; Feltenstein et al., 2009; Lynch et al., 2019; Nicolas et al., 2019), the results are equivocal when it comes to opioids, and there is very little available data concerning natural rewards like sucrose. Here, the female rats were freely cycling and estrous cycle was tracked throughout self-administration, as well as on days that they were tested for craving. There were a similar and ample number of females in nonestrus versus estrus on any given cue test day. Females self-administered increasing amounts of heroin and sucrose over the 10 d of self-administration, and there were no differences in consumption between female rats in estrus versus those in nonestrus. All female rats experienced an increase in heroin-seeking and sucrose-seeking behavior over 30 d of protracted abstinence, and there was no difference in lever pressing between estrus and nonestrus females at day 1 nor day 30. Thus, the increase in heroin and sucrose seeking cannot be attributed to a specific phase of the estrous cycle, nor is any one phase of the estrous cycle overrepresented here.

Some data indicate that female rats self-administer up to 70% less heroin during the proestrus phase of their cycle (Lacy et al., 2016), and that ovariectomized (OVX) females treated with estradiol take a greater number of heroin infusions compared with vehicle-treated OVX females (Roth et al., 2002). However, these studies were either quite short (i.e., 4 h in the case of the Lacy study), or a lower dose of heroin was used (i.e., 0.0075 mg/kg as in the case of the Roth study). In freely cycling females treated with vehicle and permitted to self-administer heroin over an extended-access 12-h session, there was no effect of estrous cycle on heroin taking (Roth et al., 2002). Other studies that used four 3-h daily sessions and a range of doses report no differences in heroin taking nor progressive ratio break point between OVX females, OVX females treated with estradiol, and males (Stewart et al., 1996; Sedki et al., 2015). Although results regarding the role of gonadal hormones on opioid self-administration are conflicting across published reports, results from this study are more consistent with other extended-access paradigms.

One recent study found that rats self-administered similar amounts of sucrose regardless of estrous cycle phase (Schmidt et al., 2021). In this article, rats were also trained on heroin self-administration before sucrose self-administration, and there was an observed decrease in heroin taking during proestrus. However, heroin self-administration and sucrose self-administration sessions were both 2 h in duration, and a lower heroin dose of 0.0075 mg/kg was used. Thus, the sucrose self-administration paradigm used was similar to the one used here, whereas the heroin self-administration paradigm was not. Ours is the first study that the authors are aware of to track estrous cycle phase alongside incubation of sucrose craving. It has been reported that there are no sex differences in sucrose seeking after 1, 21, 60, 120, and 200 d of abstinence (estrous cycle was not tracked in this study; Madangopal et al., 2019). Sucrose-related findings are extremely limited. Yet, results so far seem to be consistent with the finding that sucrose self-administration is not dependent on fluctuations in gonadal hormones.

### Locomotion is the only behavior enhanced in estrus females after 30 d of abstinence from heroin, whereas locomotion and hyperactivity are enhanced in estrus females after 30 d of abstinence from sucrose

Locomotion, sniffing, grooming, and hyperactivity were also assessed in the context of the estrous cycle. Estrus females showed increased locomotion following extended abstinence from both reinforcers. Sniffing and grooming were affected similarly in estrus versus nonestrus females at both abstinence time points for both reinforcers. Interestingly, although hyperactivity did not change across protracted abstinence in sucrose-exposed females, it was higher in estrus females at day 30. Hyperactivity increased across over time in heroin-exposed females, but was not affected by estrous cycle phase. As with data regarding the effects of the estrous cycle on incubation of craving, there was a sizeable and comparable number of estrus and nonestrus females in any given group. Thus, the fact that males and females showed largely similar behavioral profiles in early and late abstinence is unlikely to be attributable to females being tested in a specific estrous cycle phase.

The overall increase in locomotion seen in estrus females builds on previous results indicating a general increase in activity in female rats in estrus (Giles et al., 2010) by demonstrating that this occurs reliably even after treatment with either sucrose or heroin. Other studies examining stimulant-induced behavioral changes show that estrus females demonstrated reduced cocaine-induced and amphetamine-induced locomotion and increased amphetamine-induced stereotypies than those in nonestrus (Becker and Cha, 1989; Quiñones-Jenab et al., 1999). This is opposite from what was observed here in response to cues only: estrus females show an increase in locomotion, and no differences in other stereotyped behaviors. Interestingly, freely cycling females show cocaine-induced locomotor sensitization in response to cocaine challenge that is not seen in OVX females; however, OVX females treated with estradiol showed enhanced stereotyped behaviors and OVX females treated with progesterone showed reduced stereotyped behaviors (Souza et al., 2014). In these experiments, cocaine was on board. Here, we build on these previous results by demonstrating that, not only is overall locomotion increased following extended abstinence, it appears to be further enhanced in estrus females. Moreover, the increase in hyperactivity associated with heroin exposure is not attributable to the female estrous cycle since only sucrose-exposed estrus females show enhanced hyperactivity.

In conclusion, cue-induced incubation of craving occurred similarly across 30 d of extended abstinence, regardless of sex and reinforcer. This increase in active lever pressing was associated with an increase in locomotion. Analysis of more granular behaviors revealed a drug-specific behavioral profile. Specifically, heroin exposure and extended abstinence were associated with a decrease in grooming and an increase in hyperactivity in both males and females that did not generalize to sucrose-exposed rats. Whereas increased responding on a previously reward-paired lever is suitable for assessing craving broadly (as has been the standard in the field), grooming and hyperactivity should be considered in future quantifications of opioid craving specifically. Therein, these data help to refine preclinical models of opioid relapse-like behavior. Heroin craving was also associated with an increase in 50-kHz USVs in males, but not females. This indicates that males may experience a greater degree of anticipation and/or positive-valence affective states during opioid relapse events. Analysis of the female estrous cycle revealed that heroin and sucrose self-administration increase across 10 d and are not different between estrus and nonestrus females. Similarly, all female rats showed enhanced craving after protracted abstinence, and there were no differences between estrus and nonestrus females in neither early nor late abstinence. Examination of addiction-related behaviors in the context of the estrous cycle revealed that female rats in estrus showed enhanced locomotion, regardless of whether they self-administered sucrose or heroin. Interestingly, although hyperactivity was one of the heroin-specific changes, it was also enhanced in estrus females, although only those that had self-administered sucrose. Taken together, these results are a promising foundation for identifying neural mechanisms and behavioral signals associated with opioid craving in males and females. Additionally, they suggest that potential treatments may not need to be quite as sensitive to fluctuations in gonadal hormones to be effective.

## Synthesis

Reviewing Editor: Alexxai Kravitz, Washington University in St. Louis Decisions are customarily a result of the Reviewing Editor and the peer reviewers coming together and discussing their recommendations until a consensus is reached. When revisions are invited, a fact-based synthesis statement explaining their decision and outlining what is needed to prepare a revision will be listed below. The following reviewer(s) agreed to reveal their identity: NONE.

*************

Synthesis of critical comments:

*************

1) Both reviewers note that the introduction would benefit from a more clear statement about the goals and objectives of the current study. Specifically they should address the significance of why authors chose to contrast sucrose with heroin, beyond just that sucrose is a natural reinforcer and attempt to “tease out specific profile” of behaviors.

2) The discussion should also discuss the numerous differences between heroin and sucrose, and how does this affects the authors interpretation of the underlying neural profiles that manage the craving of each of these two rewards, heroin and sucrose? For instance, Reviewer 2 notes that the parameters of the self-administration sessions (duration, reinforcer availability pattern within sessions, timeout duration) also differs. Could this explain behavioral differences as well?

3) Reviewer 1 notes that more experimental detail should be given:

a) were there differences in home cage chow (or body weight) intake between males and females during abstinence or at any point during experiment?

b) were these studies possibly also run during light cycle, possibly an interaction of stress (i.e. waking up rat/disrupting circadian rhythm) and sex hormone phase?

4) Both reviewers agreed that the designation of 50 kHz ultrasonic vocalizations as “happy” sounds is problematic. It is a measure that has been anthropomorphized, and there is no supporting data to confirm that these vocalizations are associated with positive emotional valence. This should be re-phrased.

*************

Reviewer 1

*************

102-112 - Was there goal (for sucrose) or sign tracking (for both) assessed (i.e. time spent at sucrose pellet hopper or cue light)? Would this be beneficial to interpretations?

335 - authors should provide a brief mention of the significance of the distinction between assessing reinstatement measured following extinction vs. simple abstinence. And why they chose to measure the latter.

457 - were there differences in home cage chow (or body weight) intake between males and females during abstinence or at any point during experiment?

466 - were these studies possibly also run during light cycle, possibly an interaction of stress (i.e. waking up rat/disrupting circadian rhythm) and sex hormone phase?

In general, more comment on the significance of why authors chose to contrast sucrose with heroin, beyond just that sucrose is a natural reinforcer and attempt to “tease out specific profile” of behaviors. Also, given the magnitude of the craving measurement was similar or greater for sucrose, how does this fit into the authors interpretation of the underlying neural profiles that manage the craving of each of these two rewards, heroin and sucrose?

*************

Reviewer 2

*************

The submitted manuscript replicates years of work by showing that heroin and sucrose craving incubates over time such that lever pressing during a probe test is greater following 30 days of forced abstinence as compared to responding after 1 day of forced abstinence. They show this effect very clearly and their highly powered analysis shows that this measure is replicable and reliable. The novel measures in the study include the documentation of behavioral activities of rodents undergoing the probe test, and the effect of estrus cycle on these behaviors. While the authors show that there are differences in the behavioral profiles of animals undergoing incubation testing for heroin versus sucrose, there is no real assessment of the behavioral or biological relevance of these differences. Additionally, the authors report no effect of estrus cycle. Throughout the manuscript, there is an overinterpretation of the behavioral measures recorded that is not analyzed more deeply in follow up experiments. Other specific comments are listed below:

-The introduction is well written and organized and puts the study into context effectively. However, this section would benefit from a more clear statement about the goals and objectives of the current study.

-The designation of 50 kHz ultrasonic vocalizations as “happy” sounds is problematic. It is a measure that has been anthropomorphized, and there is no supporting data to confirm that these vocalizations are associated with positive emotional valence. In the absence of a deeper analysis, is it not problematic that both males and females exhibited the incubation behavior, but only males changed their vocalizations?

-The analysis of differing behavioral profiles of heroin seeking animals versus sucrose seeking animals is merely identified as different and no deeper analysis is included to determine the significance, mechanism, or relevance of these differences.

-The differences in behavioral profiles in the study is interpreted to be a result of the differing reinforcers used. However, the parameters of the self-administration sessions (duration, reinforcer availability pattern within sessions, timeout duration) also differs. Could this explain behavioral differences as well?

## Author Response

Manuscript Instructions

- Abstract and Significance Statement is missing from the manuscript text. Please add it to the text.

Abstract and significance statement were added to the manuscript.

Synthesis of critical comments:

*************

1) Both reviewers note that the introduction would benefit from a more clear statement about the goals and objectives of the current study. Specifically they should address the significance of why authors chose to contrast sucrose with heroin, beyond just that sucrose is a natural reinforcer and attempt to “tease out specific profile” of behaviors.

The introduction has been updated with an additional paragraph describing the goals and objectives of the study.

2) The discussion should also discuss the numerous differences between heroin and sucrose, and how does this affects the authors interpretation of the underlying neural profiles that manage the craving of each of these two rewards, heroin and sucrose? For instance, Reviewer 2 notes that the parameters of the self-administration sessions (duration, reinforcer availability pattern within sessions, timeout duration) also differs. Could this explain behavioral differences as well?

We agree that the methodological differences between sucrose and heroin self-administration are a caveat to our interpretations. This limitation has been added to the discussion section.

3) Reviewer 1 notes that more experimental detail should be given:

a) were there differences in home cage chow (or body weight) intake between males and females during abstinence or at any point during experiment?

b) were these studies possibly also run during light cycle, possibly an interaction of stress (i.e. waking up rat/disrupting circadian rhythm) and sex hormone phase?

Although we did not track body weight nor food intake throughout abstinence, all rats received ad libitum food and water access. The methods section has been updated to clarify this point, as well as missing experimental details about the light cycle.

4) Both reviewers agreed that the designation of 50 kHz ultrasonic vocalizations as “happy” sounds is problematic. It is a measure that has been anthropomorphized, and there is no supporting data to confirm that these vocalizations are associated with positive emotional valence. This should be re-phrased.

Interpretations of 22-kHz and 50-kHz ultrasonic vocalizations as “stressed” or “happy” have been removed and/or updated to “positive/negative-valence affective state” or “anticipatory” (50-kHz).

Reviewer 1

*************

102-112 - Was there goal (for sucrose) or sign tracking (for both) assessed (i.e. time spent at sucrose pellet hopper or cue light)? Would this be beneficial to interpretations?

We agree that goal- and sign-tracking are interesting and important components of assessing motivation and cue reactivity. However, goal or sign tracking cannot be quantified in this set of experiments for a few reasons. Goal- and sign-tracking are traditionally measured over multiple sessions, leading to the generation of an acquisition curve and categorization into sign- or goal-tracking groups based on a number of behavioral responses (e.g., cue interactions, reward location interactions). Then, the degree of sign-tracking scores are used to predict drug taking or seeking (Chang, Krueger, and Flagel, 2022; Flagel et al., 2008; Saunders and Robinson, 2010 & 2011). To the best of our knowledge, there is not a precedent for quantifying sign- and goal-tracking from a single session. We did not video record self-administration sessions, and for that reason alone, calculating sign- and goal-tracking during acquisition to a degree consistent with other literature, is not possible here.

Additionally, and perhaps more importantly, sign- and goal-tracking are Pavlovian conditioned responses or reflexes that are most accurately studied under strictly Pavlovian conditions (that is, when the lever deflection is not required for reward delivery; Pavlov 1927). In our cue test sessions here, the cue previously served as an operant discriminative stimulus that signaled the ability of the lever to provide drug infusion. Thus, the cue and lever pressing action acquired operant stimulus-response associations. Even though the cue test is performed in extinction, the cue light and lever would still hold operant associations and the behavior directed toward them reflects the previous learning history. We do acknowledge the ability for Pavlovian and operant associations to coexist, however we would argue that this particular design is not optimal for disentangling these associations.

We agree that the question of the presence, or development, of cue-driven motivation toward drug-paired cues in intravenous self-administration experiments is an interesting one. However, we would rely on a different experimental design to address this particular question. For example, a Pavlovian design where intravenous drug delivery is preceded by a lever and light cue compound presentation acquired over the course of multiple sessions. Further, drug delivery would need to be localized in space or context to allow for the possibility of a goal-tracking conditioned response. Otherwise, drug could be presented via oral consumption when mixed with sucrose (e.g., ethanol and sucrose in Krank 2003; Tomie et al. 2003) with the sucrose delivery port serving as the goal-location. In this hypothetical experiment, sign- and goal-tracking responses to the drug-paired cues would be more clear to discern and interpret. Overall, we thank the reviewer for raising this important question. Due to the design or our study, it falls beyond the scope of this article and we are not equipped to address this question thoroughly.

335 - authors should provide a brief mention of the significance of the distinction between assessing reinstatement measured following extinction vs. simple abstinence. And why they chose to measure the latter.

Thank you for this suggestion. Incubation of craving is a translationally relevant model for studying relapse. Incubation has been reported and studied in humans (Gawin and Kleber, 1986; O’Brien et al., 1992) and rodents. In rodents, it mirrors the condition in which drug use is discontinued and then individuals reintegrate into a familiar environment after some time away (hospitalization, incarceration, in-patient rehabilitation, etc.) We have reinforced this point in the updated version of the manuscript.

457 - were there differences in home cage chow (or body weight) intake between males and females during abstinence or at any point during experiment?

All animals had ad libitum access to food and water throughout all phases of the experiment, including abstinence. Weights were not collected at any time throughout abstinence, so we are not able to address this particular question.

466 - were these studies possibly also run during light cycle, possibly an interaction of stress (i.e. waking up rat/disrupting circadian rhythm) and sex hormone phase?

Thank you for pointing this out. We agree that running animals during the light cycle would potentially disrupt circadian rhythm. All our animals were housed in a reverse light-dark colony room (lights OFF at 9:00AM), and experiments were run during the dark phase. The methods section has been updated to reflect this more clearly.

In general, more comment on the significance of why authors chose to contrast sucrose with heroin, beyond just that sucrose is a natural reinforcer and attempt to “tease out specific profile” of behaviors. Also, given the magnitude of the craving measurement was similar or greater for sucrose, how does this fit into the authors interpretation of the underlying neural profiles that manage the craving of each of these two rewards, heroin and sucrose?

We appreciate your feedback that our rationale for including a sucrose control group was unclear. Other studies often use saline as a control group when assessing behavioral and molecular changes associated with craving (Cates et al., 2018; Werner et al., 2018; and many others). However, saline does not affect the mesolimbic reward pathway in the same way as drugs of abuse. Sucrose, a natural non-drug reward, has overlapping neural substates with drugs of abuse, and yet it is non-pathological, and not the intended target of potential treatments. We aimed to identify behaviors that accompany reward seeking/craving generally. Another objective of this study was to identify drug-specific behavioral correlates of craving/seeking that do not generalize to natural rewards. Using this method, we can gain important insight into characteristics of non-pathological craving behaviors versus those that specifically accompany heroin craving. Furthermore, using sucrose allows us to differentiate the mechanisms of cue-reward associations and memory consolidation broadly from those that govern opioid-paired cue-induced craving.

These findings lay the groundwork for investigating neural mechanisms and drug-induced changes that lead to the development of this distinct behavioral signature. An in-depth explanation of specific mechanisms has not been fully elucidated and is beyond the scope of this article. However, we hypothesize that the pathways involved in opioid craving are at least partially distinct from those that drive craving/seeking more broadly for other reinforcers, including sucrose. Some potential mechanisms are explored in the following sub-sections of the discussion: “Increase in lever pressing is associated with increased locomotion in males and females, regardless of reinforcer” and “In both sexes, chronic heroin exposure and extended abstinence results in an opioid-specific behavioral profile”.

As for the magnitude of craving being similar across reinforcers/higher in sucrose-exposed animals, this highlights the point that cue-reward associations intensify throughout abstinence, regardless of reinforcer. These results suggest that memory/systems consolidation and related neural underpinnings drive this intensification similarly across reinforcers. Though we did not probe specific circuits or molecular correlates here, we hope that the clear and thorough signatures described here lay the groundwork for these sorts of studies.

Reviewer 2

*************

-The introduction is well written and organized and puts the study into context effectively. However, this section would benefit from a more clear statement about the goals and objectives of the current study.

Thank you for your suggestion. We added the abstract, significance statement, and updated the introduction accordingly.

-The designation of 50 kHz ultrasonic vocalizations as “happy” sounds is problematic. It is a measure that has been anthropomorphized, and there is no supporting data to confirm that these vocalizations are associated with positive emotional valence. In the absence of a deeper analysis, is it not problematic that both males and females exhibited the incubation behavior, but only males changed their vocalizations?

Thank you for this comment. We agree that “happy” is problematic and anthropomorphic. We have corrected “happy” and “stressed” to “positive-valence” and “negative-valence affective state” and/or removed these phrases throughout. Our interpretation of 50-kHz ultrasonic vocalizations is based on previous literature suggesting they are linked to positive affective states. Other studies also interpret 50-kHz USVs as positive-valence affective states in anticipation of drug reward (Buck et al., 2014; Ma et al., 2010). Our interpretation of 22-kHz USVs is based on previous literature, which suggests they are related to negative affective states (Williams et al., 2012). They are often termed “distress” calls (Mutschler & Miczek, 1998). Ultrasonic vocalizations are meant to serve communicative functions so they can unequivocally communicate the state of the animal making the call to a conspecific (Brudzynski, 2013). Therefore, 22- and 50-kHz ultrasonic vocalizations have at least some levels of valence discernable to conspecifics. This was the rationale for our interpretation of them as positive/negative-valence affective states. Per your suggestion, we have updated the language to reflect those characterizations as opposed to the more human “happy"/"stressed”.

It is interesting that males and females exhibited incubation behavior, but males only changed their vocalizations. We hypothesize that males emit 50-kHz USVs as an anticipatory behavior during cue re-exposure. This is consistent with other reports that males relapse during anticipatory/positive mood states, whereas females report greater negative affect prior to cocaine and alcohol relapse (McKay et al., 1996; Rubin et al., 1996). Females also report a greater increase in sadness and decrease in joy upon exposure to opioid-paired imagery and paraphernalia (Yu et al., 2007). This may help explain why both sexes showed relapse-like behavior (increased responding during the cue test), but only males emitted 50-kHz USVs. This interpretation has been added to the discussion.

-The analysis of differing behavioral profiles of heroin seeking animals versus sucrose seeking animals is merely identified as different and no deeper analysis is included to determine the significance, mechanism, or relevance of these differences.

We appreciate this point and have added this caveat to the discussion section. The mechanism for explaining the different behavioral profiles in heroin- versus sucrose-exposed rats is undetermined and outside the scope of this report. Ultimately, we are hoping that these descriptive data lay the foundation for further exploration of these mechanisms. Most of the research on incubation of craving has utilized responses on a previously reward-paired lever to assess craving, regardless of reinforcer. Here, we show that other behaviors, such as grooming and hyperactivity, may be better suited for quantifying opioid craving specifically. We believe our behavioral profiles are significant and relevant because they 1) underscore the wealth of data indicating that increased active lever presses occur in males and females upon cue re-exposure after prolonged abstinence, while 2) adding more nuanced and robust measurements of opioid craving specifically. These data can help improve preclinical models of craving/relapse-like behavior while also serving as a powerful foundation for additional mechanistic exploration.

-The differences in behavioral profiles in the study is interpreted to be a result of the differing reinforcers used. However, the parameters of the self-administration sessions (duration, reinforcer availability pattern within sessions, timeout duration) also differs. Could this explain behavioral differences as well?

Thank you for pointing this out, your point is well taken. As noted, there is no way to be certain that differences in session length and continuous versus intermittent access do not generate the behavioral changes seen here. This is a limitation of the study and has been added to the discussion.

We utilized a long-access heroin self-administration paradigm because there is evidence that this is necessary for incubation of craving (Salisbury, Blackwood, and Cadet, 2021), whereas previous studies find no differences in incubation of sucrose craving given 2 or 6 hours of access (Grimm, Fyall, and Osincup, 2005). Regardless of session length, both the heroin- and sucrose-exposed rats increased their intake over 10 days. We have further highlighted these important caveats in the updated discussion of the manuscript.
